# Trimodal Therapy vs. Radical Cystectomy for Muscle-Invasive Bladder Cancer: A Meta-Analysis

**DOI:** 10.3389/fonc.2020.564779

**Published:** 2020-10-14

**Authors:** Hui Ding, Ning Fan, Zhongyun Ning, Deyuan Ma

**Affiliations:** ^1^Key Laboratory of Diseases of Urological System Gansu Province, Department of Urology, Gansu Nephro-Urological Clinical Center, Lanzhou University Second Hospital, Lanzhou, China; ^2^Department of Urology, University of Pittsburgh School of Medicine, Pittsburgh, PA, United States; ^3^The Second Clinical College of Lanzhou University, Lanzhou, China

**Keywords:** muscle-invasive bladder cancer, bladder cancer, trimodal therapy, radical cystectomy, meta-analysis

## Abstract

**Background:** To compare the difference between trimodal therapy (TMT) and radical cystectomy (RC) in treating muscle-invasive bladder cancer, we performed a meta-analysis for data from the following database.

**Methods:** We searched PubMed, Chinese biomedicine literature database, the Cochrane Library, China National Knowledge Internet databases, Wanfang databases, and Google Scholar up to December 2019. The main outcome measures assessed were overall survival (OS), cancer-specific survival (CSS), mortality, and Charlson comorbidity score (CCS). Two authors independently evaluated the study quality and extracted data. All data were analyzed using Review Manager (version 5.3).

**Results:** After database retrieval, article selection, data extraction, and quality assessment, nine articles comprising 5,721 cases from the TMT group and 48,262 cases from the RC group were included in this study. The data showed that there was no statistical difference between TMT and RC at <10 years OS [pooled hazard ratio (HR) = 1.26, 95% confidence interval (CI): 0.92–1.73, *Z* = 1.46, *P* = 0.14], while OS of the RC group was higher than that of the TMT group at more than 10 years (pooled HR = 1.34, 95% CI: 1.18–1.54, *Z* = 4.33, *P* < 0.0001). As for CSS, compared with the TMT group, the patients in the RC group had longer CSS (pooled HR = 1.50, 95% CI: 1.29–1.76, *Z* = 5.15, *P* < 0.00001). Compared with RC, TMT is linked to an obvious increase in all-cause mortality and bladder-specific cancer mortality (pooled HR = 1.30, 95% CI: 1.16–1.46, *Z* = 4.55, *P* < 0.00001; pooled HR = 1.32, 95% CI: 1.15–1.51, *Z* = 3.92, *P* < 0.0001). The bladder cancer patients belonging to CCS “0” score preferred RC [pooled relative risk (OR) = 0.94, 95% CI: 0.89–0.98, *Z* = 2.79, *P* = 0.005], while CCS “2” score's patients were prone to TMT (pooled OR = 1.40, 95% CI: 1.29–1.53, *Z* = 7.73, *P* < 0.00001).

**Conclusions:** Overall, this meta-analysis suggests that the efficacy of TMT is non-inferior to that of RC at <10-year OS, and RC is superior to TMT at more than 10-year OS. Therefore, TMT may be a reasonable treatment option in well-selected patients who are unsuitable for surgery or are not willing to experience surgery. In the future, more high-quality, large-sample randomized controlled trials (RCTs) are needed to verify the results.

## Introduction

Bladder cancer is one of the most common urinary tract tumors in the US, with an estimated 80,500 new cases and 17,600 deaths in 2019 (1). Patients with advanced and metastatic bladder cancer had lower survival rates, with 5-year survival rates of 34% in localized disease, 7% in regional disease, and 5% in metastatic disease.

Radical cystectomy (RC) plus pelvic lymph node dissection is commonly regarded as the gold standard therapy for muscle-invasive bladder cancer (MIBC) (2–4). But some patients have a strong willingness to preserve their own bladders, and bladder-sparing becomes their preferred option. The existing bladder preservation approaches are partial cystectomy, transurethral resection (TUR) alone, single chemotherapy, or radiation therapy (RT). It is generally considered that monotherapy is inferior to RC for MIBC.

Recently, a trimodal therapy (TMT), which includes utmost TUR followed by simultaneous chemotherapy and RT, is the most effective strategy for preserving the bladder (5, 6). Several studies reported that for overall survival (OS), the effects of TMT on MIBC were parallel to RC (7, 8). A published series systematic review indicated that TMT results in satisfactory outcomes and may be a rational therapy option in well-selected patients (9). Furthermore, several clinical trials compared the outcomes between RC and TMT (10–12). Most of the literature included in the previous meta-analysis were case series (9, 13, 14) and did not directly compare the efficacy of RC and TMT; and not all included patients were performed standard TMT in the other meta-analysis (15). So, it is essential to perform a meta-analysis directly comparing RC and standard TMT. The aim of this meta-analysis was to evaluate the difference in OS and other outcome indicators after using either of the two treatment modalities in MIBC because we believe that accumulating evidence from studies should be more reliable.

## Methods

### Search Strategy

We searched Pubmed (1966–December 2019), Chinese biomedicine literature database (1978–December 2019), and the Cochrane Central Register of Controlled Trials via the Cochrane Library on December 2019. The China National Knowledge Internet databases, Wanfang databases, and Google Scholar were also retrieved. Search terms combined patient-related terms (bladder cancer) and intervention terms (bladder preservation or organ-sparing or bladder-sparing or trimodality treatment or radiotherapy or chemotherapy or chemoradiation or chemoradiotherapy or cystectomy).

### Inclusion Criteria and Study Eligibility

We estimated the records on the basis of the Preferred Reporting Items for Systematic reviews and Meta-Analysis statement. We defined study eligibility using the PICO (patient population, intervention, comparator, and outcomes) and setting methods. Included studies were those that compared patient outcomes between TMT and RC in MIBC patients. The searches were performed in written English or Chinese. Published clinical controlled studies and randomized controlled trials (RCTs) were included. When two or more studies were reported by the same institution and/or authors in overlapping time periods, the most recently published report that included the largest number of patients was used.

### Data Extraction

**Figure 1:**
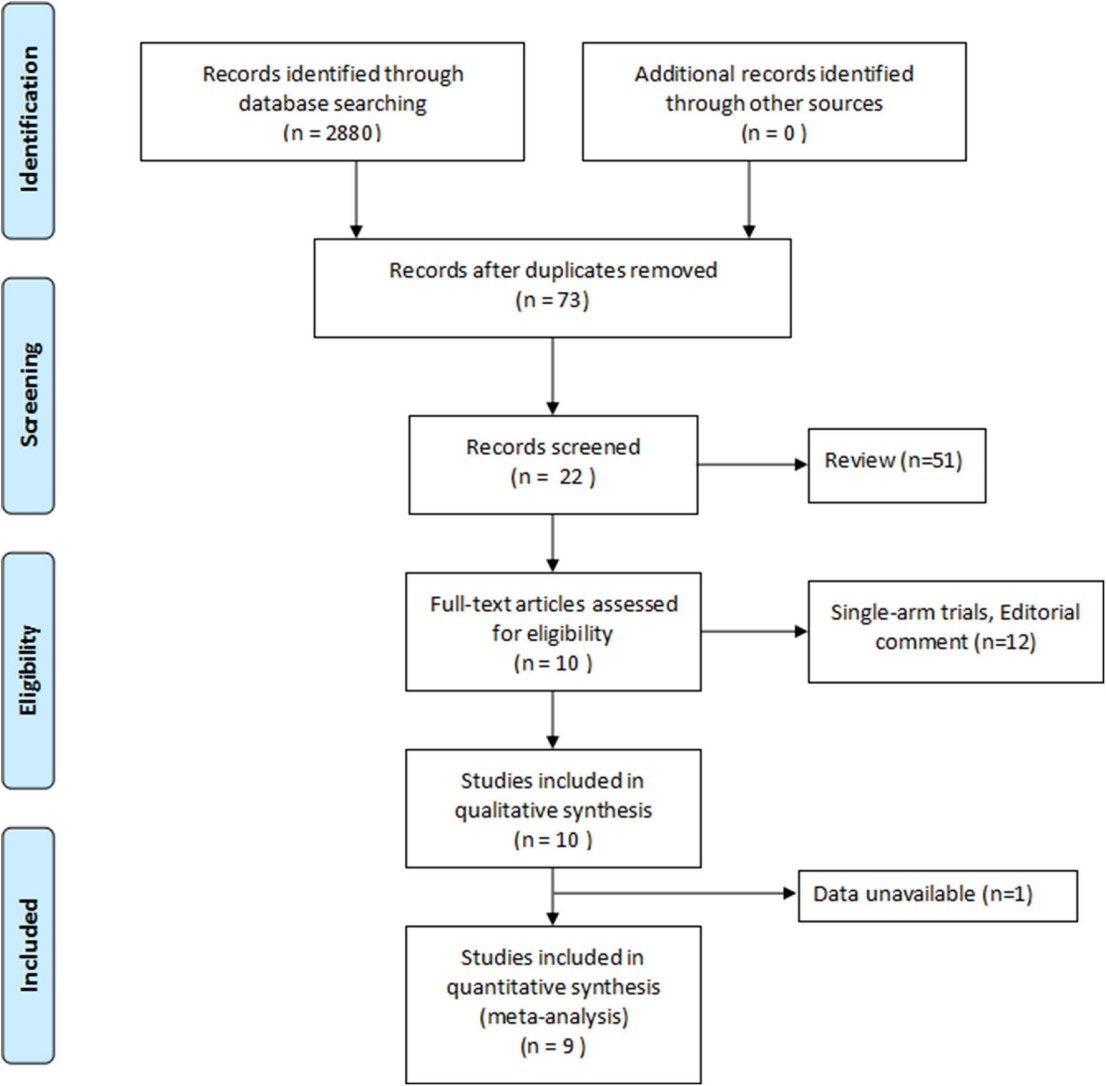
Flowchart of meta-analysis.

Data extraction was performed independently by the same authors using standard data extraction forms. Disagreements were resolved in consultation with the third reviewer. For each study, we collected the following characteristics: name of the first author, year of publication, ethnicity, and country of study population. Primary outcomes included OS, cancer-specific survival (CSS), mortality, and Charlson comorbidity score (CCS) after TMT or RC treatment. Screening of articles is shown in a flowchart (Figure 1). When studies included article type of >1, data were extracted separately based on categories for sensitivity analyses.

### Statistical Analysis

Log hazard ratio (HR) and the variance were used as the summary outcome measure from all trials in the meta-analysis. For each study, we derived the HR at the 95% confidence interval (CI) of data to assess the difference between TMT and RC. The OS, CSS, CCS, Eastern Cooperative Oncology Group (ECOG) score, and clinical T stage of patients with TMT or RC were also compared by odds ratio (OR) or Hazard ratio (HR) with 95% CI. The *Z*-test was employed to determine the statistical significance of the summary OR. *I*^2^-test and chi-square test were employed to evaluate the heterogeneity among the studies. If *P* <0.10, it was considered to have significant heterogeneity in statistics; and the *I*^2^ value was employed to detect the degree of heterogeneity (*I*^2^ <25%, no heterogeneity; *I*^2^ = 25–50%, moderate heterogeneity; *I*^2^ > 50%, large or extreme heterogeneity). To test the reliability of the results, the fixed-effects (Mantel–Haenszel method) and the random-effects (DerSimonian–Laird method) models were used to assess the pooled OR and HR, respectively. Ethical approval was not required for this study as it was a study using systematic review and meta-analysis. The quality of included studies was evaluated using the methodological index for non-randomized studies (MINORS), with 0 indicating the lowest and 24 as the highest score (16).

Review Manager, version 5.3, software was used to perform the meta-analyses (The Cochrane Information Management System, http://ims.cochrane.org/revman). *P* < 0.05 was considered statistically significant.

## Results

### Eligible Studies

A total of 2,880 records were acquired by searching the six databases. By removal of duplicates, reviews, and not relevant to the question, 22 articles remained. Then, after screening the full text of these articles, nine articles (10–12, 17–22) were assessed for eligibility. Further evaluations and detailed analysis of the articles were illustrated in Figure 1.

### Literature Analysis

The meta-analysis included 53,983 bladder cancer patients, with 5,721 from the TMT group and 48,262 from the RC group. All studies were published in English, and retrospective controlled observational studies and no RCTs were found. The OS was directly reported in seven studies, three studies reported the data of CSS, and two studies reported the mortality. All the details of study characteristics are summarized in Table 1. The MINORS is from 16 to 19 in the included studies (Table 2), which are viewed as moderate to high quality.

**Table 1:** The Main Characteristics of Included Studies.

**Author**	**Patients**	**Gender**	**Age**	**Clinical T stage**	**Cancer grade**	**ECOG Score**	**CCS (%)**	**Chemoradiotherapy**
**(year)**	**(*n*)**	**Male vs. Female**	**(median or mean)**	**≤2 vs. >2(%)**	**UC vs. Other**	**0 vs. ≥1 (%)**	**0 vs. 1 vs. ≥2 (%)**	
(1)							Unclear	Cisplatin:100, 200, or 300 mg
RC	62	48 vs. 14	64	50 vs. 50	56 vs. 6	46.8 vs. 53.2		
TMT	62	43 vs. 19	72	24.2 vs. 75.8	55 vs. 7	33.9 vs. 66.1		Radiation:60.4 Gy
(20)				Unclear	Unclear	Unclear	Unclear	Unclear
RC	1,426	892 vs. 534	75.4					
TMT	417	300 vs. 117	79.3					
(12)						Unclear	Unclear	Gemcitabine:1,000 mg/m^2^
RC	308	260 vs. 48	65	47.1 vs. 52.9	308 vs. 0			Cisplatin:70 mg/m^2^
TMT	32	25 vs. 7	77	56.3 vs. 43.8	32 vs. 0			Radiation:46 Gy
(11)			71		Unclear		≤ 4 vs. >4	Cisplatin: 40 mg/m^2^
RC	56	41 vs. 15		73.2 vs. 26.8		40 vs. 16	69.6 vs. 30.4	Radiation:66 Gy
						40 vs. 16	66.1 vs. 33.9	
TMT	56	40 vs. 16		67.9 vs. 32.1				
(19)			<60 vs. ≥60(%)			Unclear	0 vs. 1 vs. ≥2	Any chemotherapy
RC	22,680	17,055 vs. 5,625	19.9 vs. 80.1	54.2 vs. 45.8	20,503 vs. 2,177		70 vs. 22.9 vs. 7.1	Radiation:50–80 Gy
TMT	1,489	1,112 vs. 377	5.8 vs. 94.2	81.9 vs. 18.1	1,330 vs. 159		66.8 vs. 22.8 vs. 10.4	
(10)			Mean		Unclear	Unclear	0 vs. 1 vs. ≥2	Any chemotherapy
RC	11,586	8,725 vs. 2,861	68.1	80.1 vs. 19.9			70.3 vs. 23 vs. 6.7	Radiation:60–65 Gy
TMT	1,257	955 vs. 302	74.8	82.1 vs. 17.9			68.5 vs. 23.3 vs. 8.2	
(17)						Unclear	0 vs. 1 vs. ≥2	Any chemotherapy
RC	7,276	5,499 vs. 1,777	67.39	86.48 vs. 13.52	7,276 vs. 0		69.46 vs. 23.67 vs. 6.87	Radiation:64.8 Gy
TMT	1,178	863 vs. 315	75.21	88.71 vs. 11.29	1,178 vs. 0		65.62 vs. 24.96 vs. 9.42	
(22)			75.8			Unclear	0 vs. 1 vs. ≥2	Cisplatin or fluorouracil and mitomycin C
RC	2,448	1,516 vs. 932		39.5 vs. 60.5	2,387 vs. 61		56.6 vs. 26.4 vs. 17	
TMT	752	532 vs. 220		70.7 vs. 29.3	709 vs. 43		47.1 vs. 27.4 vs. 25.5	Radiation:60–66 Gy
(18)					Unclear	Unclear	Unclear–	Unclear
RC	2,420	1,611 vs. 809	65.0	21.6 vs. 78.4				
TMT	478	359 vs. 119	67.5	63.2 vs. 36.8				

*ECOG, Eastern Cooperative Oncology Group; CCS, charlson comorbidity score; TMT, trimodal therapy*.

**Table 2:** The MINORS score of Included Studies.

**Methodological item for non-randomized studies**	**(1)**	**(20)**	**(12)**	**(11)**	**(19)**	**(10)**	**(17)**	**(22)**	**(18)**
1. A clearly stated aim	2	2	2	2	2	2	2	2	2
2.Inclusion of consecutive patients	2	2	2	2	2	1	2	2	1
3.Prospective collection of data	1	1	1	1	1	1	1	1	1
4.Endpoints appropriate to the aim of the study	1	1	1	1	1	1	1	1	1
5.Unbiased assessment of the study endpoint	1	1	1	1	1	1	1	1	1
6.Follow-up period appropriate to the aim of the study	2	2	2	2	2	2	2	2	2
7.Loss to follow up <5%	2	2	2	2	2	2	2	2	1
8.Prospective calculation of the study size	0	0	0	0	0	0	0	0	0
9.An adequate control group	2	2	2	2	2	2	2	2	2
10.Contemporary groups	2	2	2	2	2	2	2	2	2
11.Baseline equivalence of groups	0	0	0	2	0	2	0	0	1
12.Adequate statistical analyses	2	2	2	2	2	2	2	2	2

**Figure 2:**
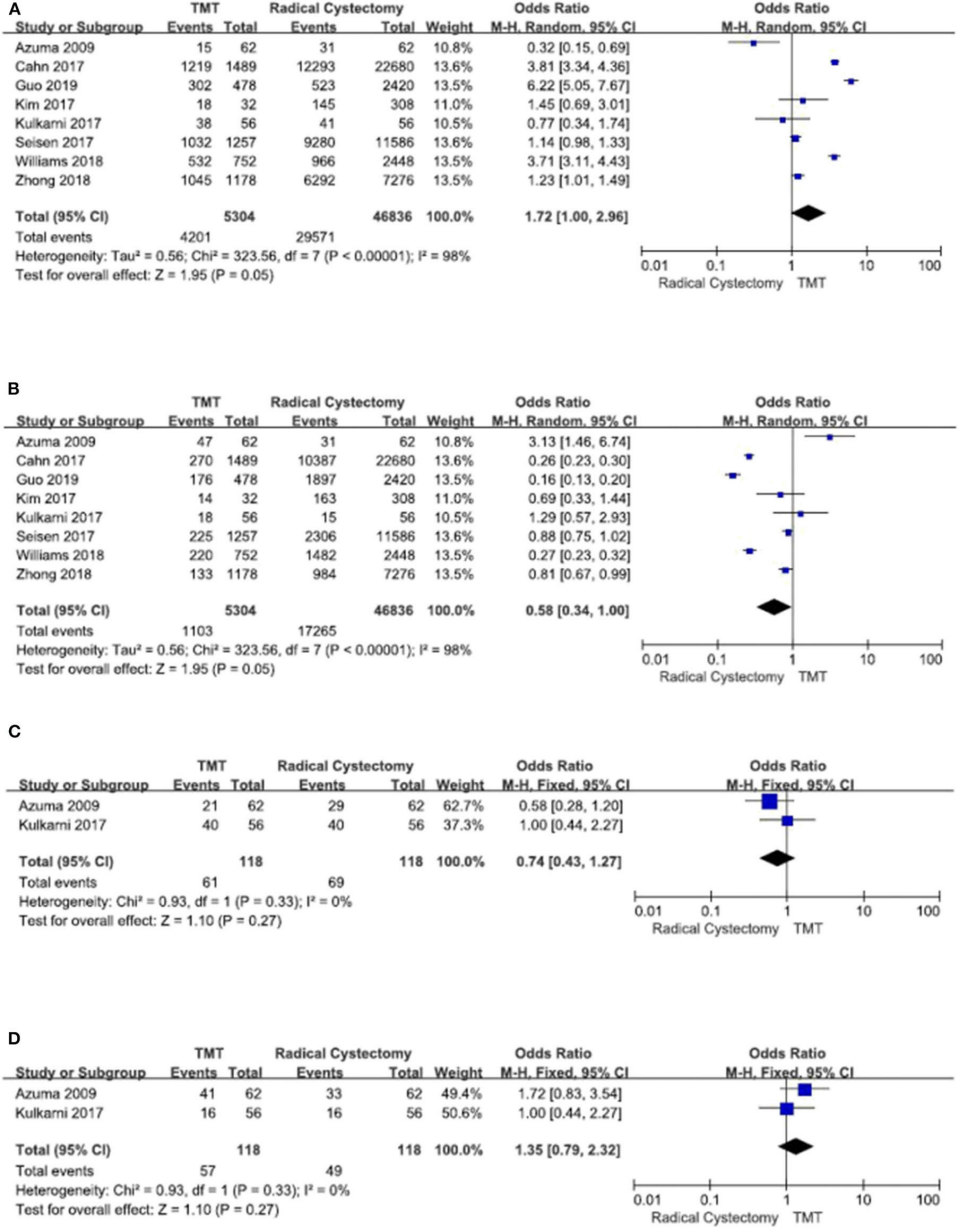
Forest plot comparing clinical T stage and Eastern Cooperative Oncology Group (ECOG) in patients receiving trimodal therapy (TMT) vs. radical cystectomy (RC). **(A)** T stage ≤ 2, **(B)** T stage > 2, **(C)** ECOG = 0, and **(D)** ECOG ≥ 1.

From the pathology grade, most of the included patients have urothelial carcinoma, and other patients have adenocarcinoma, squamous cell carcinoma, choriocarcinoma, and unknown. From clinical T stage and ECOG score, there was no obvious difference between the two groups (Figure 2).

### Meta-Analysis

#### Overall Survival

**Figure 3:**
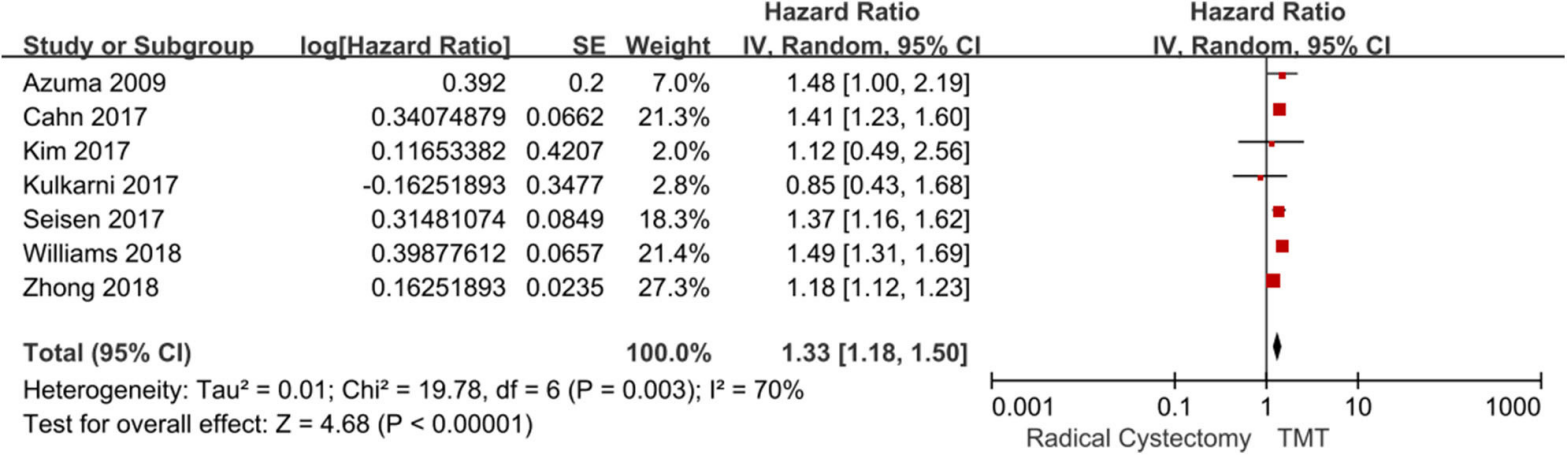
Forest plot comparing overall survival in patients receiving trimodal therapy (TMT) vs. radical cystectomy (RC).

**Figure 4:**
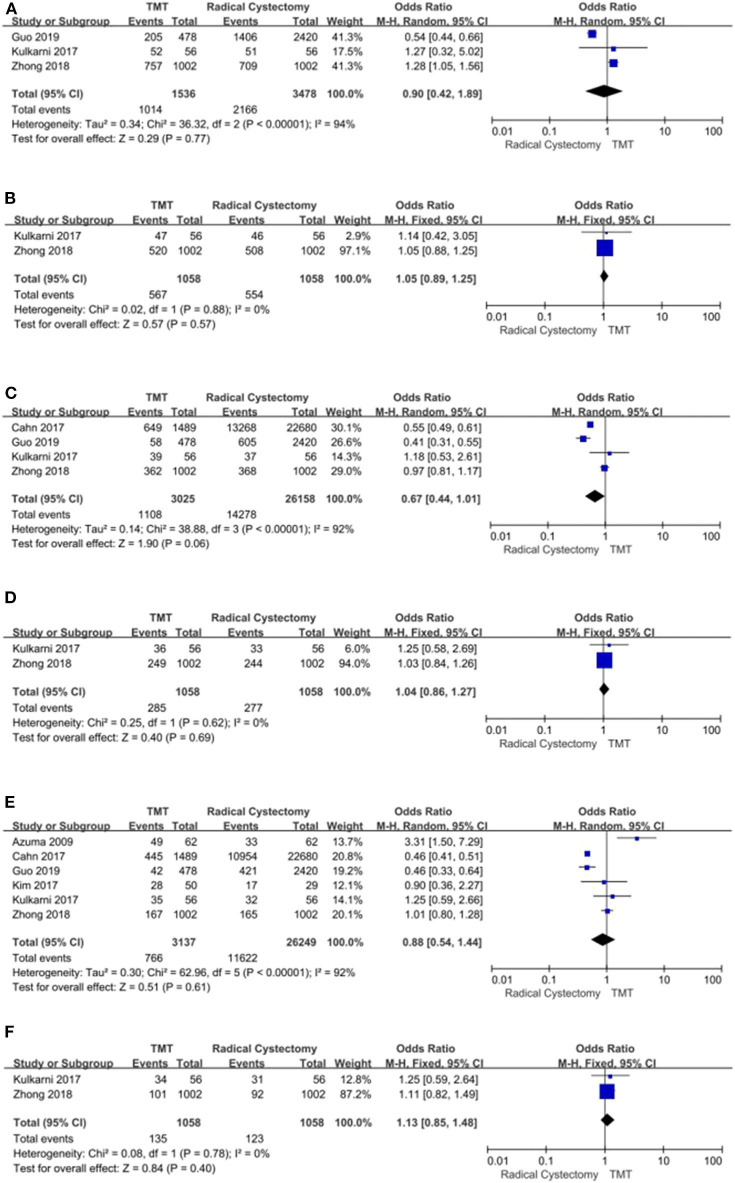
Forest plot comparing overall survival in patients receiving trimodal therapy (TMT) vs. radical cystectomy (RC) at 12 **(A)**, 24 **(B)**, 36 **(C)**, 48 **(D)**, 60 **(E)**, and 72 **(F)** months.

Seven studies compared the OS between TMT and RC. Since there was obvious heterogeneity among these studies (*I*^2^ = 70%, *P* = 0.003), the random-effects model was used to calculate the pooled HR. The data showed that the OS of the RC group was higher than that of the TMT group (pooled HR = 1.33, 95% CI: 1.18–1.50, *Z* = 4.68, *P* < 0.00001, Figure 3). According to the follow-up time, the pooled HR results showed that there was no statistical difference between TMT and RC at <10 years (pooled HR = 1.26, 95% CI: 0.92–1.73, *Z* = 1.46, *P* = 0.14), and the pooled OR results also showed that there was no statistical difference between TMT and RC at 12, 24, 36, 48, 60, and 72 months, respectively (Figure 4); however, the OS of the RC group was higher than that of the TMT group at more than 10 years (pooled HR = 1.34, 95% CI: 1.18–1.54, *Z* = 4.33, *P* < 0.0001).

#### Cancer-Specific Survival

**Figure 5:**
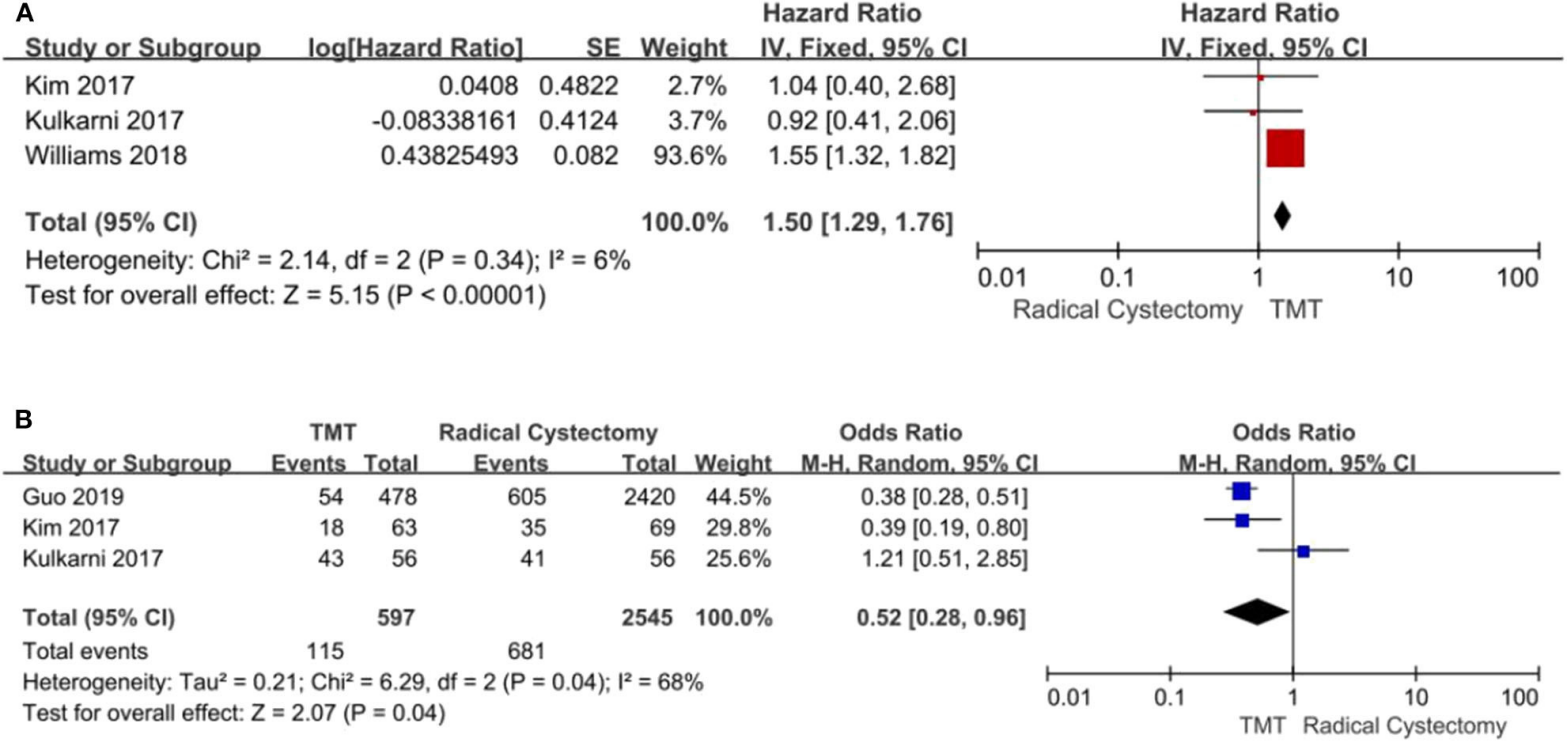
Forest plot comparing cancer-specific survival (CSS) in patients receiving trimodal therapy (TMT) vs. radical cystectomy (RC). **(A)** Pooled hazard ratio (HR); **(B)** Pooled odds ratio (OR).

Three studies with 3,652 patients enrolled reported the CSS regarding both TMT and RC. The fixed-effects model was chosen to assess the combined RR for no significant heterogeneity among the studies (*I*^2^ = 6%, *P* = 0.34). The results demonstrated that compared with the TMT group, the patients in the RC group had longer CSS (pooled HR = 1.50, 95% CI: 1.29–1.76, *Z* = 5.15, *P* < 0.00001, Figure 5A; pooled OR = 0.52, 95% CI: 0.28–0.96, *Z* = 2.07, *P* = 0.04, Figure 5B).

#### Mortality

**Figure 6:**
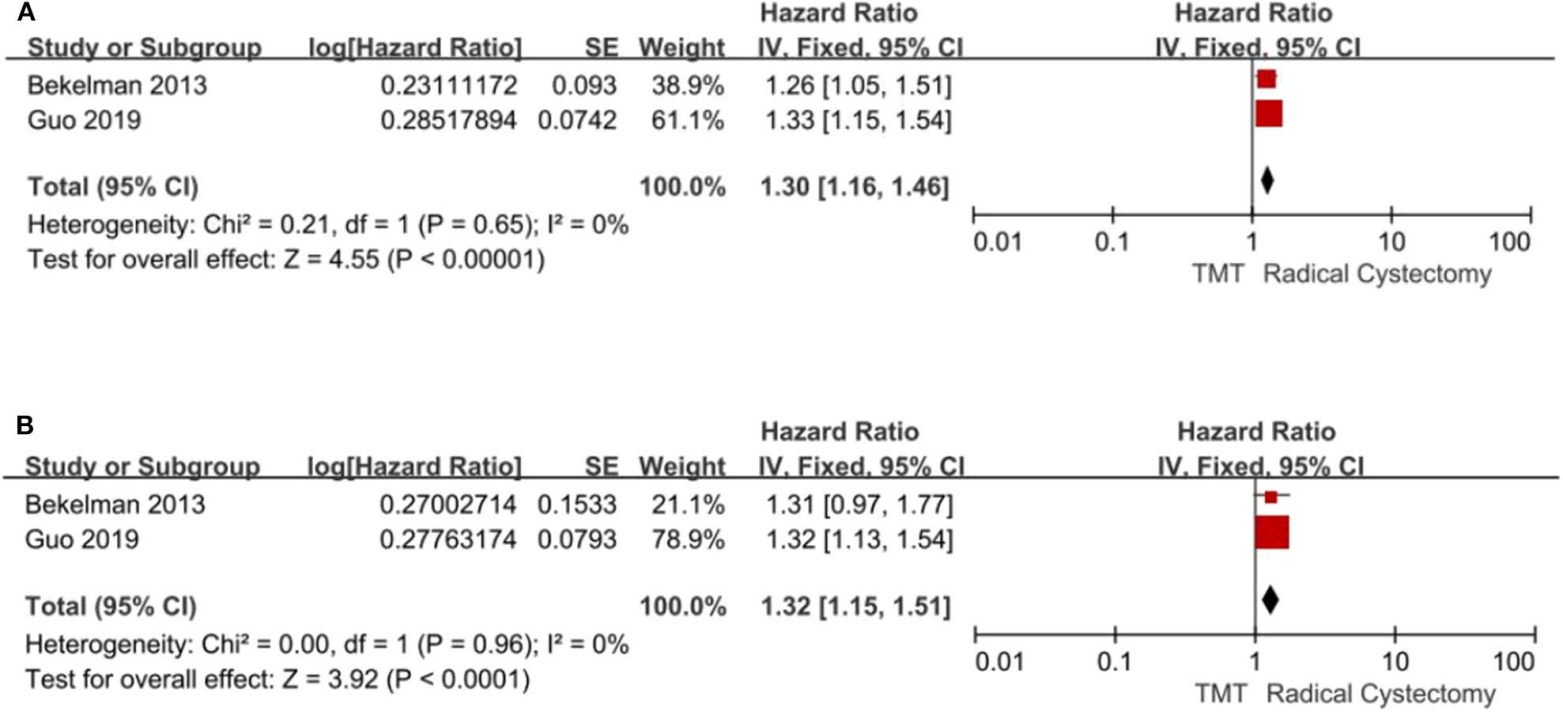
Forest plot comparing mortality in patients receiving trimodal therapy (TMT) vs. radical cystectomy (RC). **(A)** All-cause mortality; **(B)** bladder-specific cancer mortality.

Two studies compared all-cause mortality and bladder-specific cancer mortality between TMT and RC, respectively. The pooled HR results showed that compared with RC, TMT is associated with a significant increase in all-cause mortality and bladder-specific cancer mortality (pooled HR = 1.30, 95% CI: 1.16–1.46, *Z* = 4.55, *P* < 0.00001, Figure 6A; pooled HR = 1.32, 95% CI: 1.15–1.51, *Z* = 3.92, *P* < 0.0001, Figure 6B).

#### Charlson Comorbidity Score

**Figure 7:**
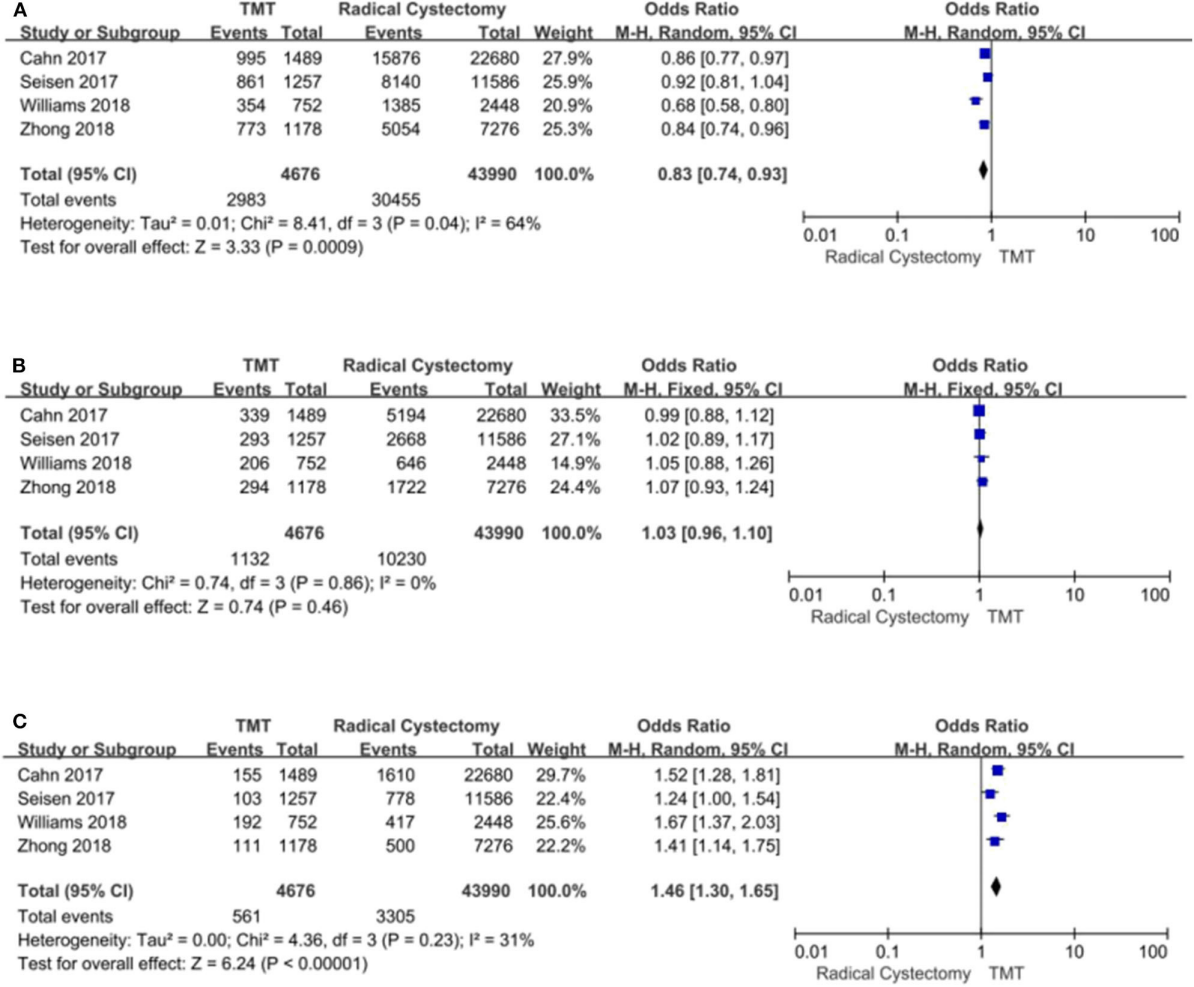
Forest plot comparing Charlson comorbidity score (CCS) in patients receiving trimodal therapy (TMT) vs. radical cystectomy (RC). **(A)** CCS = 0; **(B)** CCS = 1; **(C)** CCS ≥ 2.

According to stratified analysis of the CCS score, the bladder cancer patients belonging to CCS “0” score preferred RC (pooled OR = 0.83, 95% CI: 0.74–0.93, *Z* = 3.33, *P* = 0.0009, Figure 7A), and there were no significant differences in CCS “1” score's patients between TMT and RC Figure 7B, while CCS “2” score's patients were prone to TMT (pooled OR = 1.46, 95% CI: 1.30–1.65, *Z* = 6.24, *P* < 0.00001, Figure 7C).

### Sensitivity Analysis

A sensitivity analysis was used to examine the OS result stability. The sensitivity analysis showed that each individual study could not affect the final pooled results. This indicates the robustness and constancy of the results.

## Discussion

Since there is a lack of RCTs directly comparing RC and bladder preservation therapy with TMT, and the previous systematic review mainly focused on case series or incomplete TMT, this study uses the meta-analysis method to analyze the effects of both complete TMT and RC on MIBC from available clinical controlled trials. The data suggest that compared to complete TMT patients, RC patients have a higher overall OS, CSS, and less mortality for MIBC patients. According to the stratified analysis, it was found that there was a statistically significant difference at more than 10-year OS between the two groups. Moreover, the results demonstrated that the bladder cancer patients belonging to CCS “0” score preferred RC, while CCS “2” score's patients were prone to TMT.

Previous meta-analysis studies have shown inconsistencies for TMT and RC. Arcangeli et al. (14) reported that TMT can generate outstanding 5-year OS rates between these two interventions. Fahmy et al. (13) indicated that the OS and disease-specific survival (DSS) were comparable between TMT and RC: the average 10-year OS rate was 30.9% for TMT and 35.1% for RC, respectively (*P* = 0.32), and the average 10-year DSS rate was 50.9% for TMT and 57.8% for RC, respectively (*P* = 0.26). García-Perdomo et al. (15) reported that CSS rate favored patients who underwent RC. However, most included studies were case series in the systematic reviews by Arcangeli et al. (14) and Fahmy et al. (13). In the study by García-Perdomo et al. (15), some studies included only chemoradiotherapy that was not the standard TMT. Ploussard et al. (9) only included patients receiving TMTs.

It is reported that the 5-year OS was from 50 to 60% in the literature. The Radiotherapy Oncology Group (RTOG) has completed six prospective TMT regimens for MIBC patients undergoing cystectomy. Five of the RTOG regimens are phase I–II trials for simultaneous chemotherapy and radiotherapy, and one is a phase III trial to test the efficacy of adjuvant chemotherapy with methotrexate, cisplatin, and vinblastine. A total of 415 patients were entered on these trials. The 5-year OS rate was near to 50%, with 75% of those patients accomplishing a cure for their bladder cancer while maintaining bladder function (23). In the most recently published long-term follow-up of 348 patients from the Massachusetts General Hospital (MGH) in the USA, they show that 5-year OS rates and CSS rates were 52 and 64% for split-course TMT, respectively (24). Another continuous-course TMT series comes from Erlangen, Germany. A total of 331 patients were permanently followed up, and the overall 5-year OS rate was 54% (25). Compared to TMT, RC has been reported in previous studies to show comparable outcomes. These studies have reported that 5-year OS rates were 58–68% in patients receiving primary RC (26–28). Our meta-analysis showed that the longest follow-up time was <10 years in three studies, and no difference was found between them at <10 years' OS and CSS.

However, the final follow-up time was more than 10 years in the five studies; RC was superior to TMT at more than 10 years' OS and CSS. Faraj et al. (29) reported that the 10-year OS was 45.6% for RC patients. For bladder-preserving combined-modality therapy, the 10-year OS rates were 36–39% (7, 30).

The adverse effects of RC were associated with sexual dysfunction and external urinary drainage devices, which cause emotional and psychological stress. However, two prospective trials have confirmed that bladder-sparing must be beneficial to improve quality of life (QOL) after TMT compared to RC (31, 32). TMT was reported to be associated with better sexual function (*P* < 0.02) and better body image perception (*P* < 0.001) compared to RC; TMT had better general QOL compared with those who had received RC (*P* = 0.001) and higher physical, role, social, emotional, and cognitive functioning (*P* ≤ 0.04); TMT was associated with better bowel function (*P* = 0.02) and fewer bowel symptoms (*P* ≤ 0.05) (33).

Although the patients are afraid of TMT's toxicity, studies show that it is acceptable for patients with bladder preservation treatment. The main acute toxicities include hematologic, gastrointestinal (GI), and genitourinary (GU). In the Bladder Cancer 2001 trial (6), 182 patients underwent chemoradiotherapy and median follow-up was 69.9 months; the results indicated that there was slightly increased acute grade 3 or 4 adverse events (AEs) in the chemoradiotherapy group, and these events were mostly GI toxic effects.

The proportion of late grade 1–2 toxicity was from 6 to 25% for GU and 5 to 6% for GI toxicities, respectively (34–37). Among which, the primary low-grade toxicities included urgency, nocturia, dysuresia, incontinence, hemorrhagic cystitis, diarrhea, and rectitis. The percentage of late grade 3 urinary tract toxicity was from 3 to 8% of the series (6, 38, 39). In the BC2001 trial, there were no differences in late toxicity after adding chemotherapy to RT; of these, the grade 3–4 toxicity rate was 0.8% for GI symptoms and 7.4% for GU symptoms, respectively (6).

Based on these studies, it is clear that TMT is safe and effective for bladder cancer patients without serious side effects. However, we should note that TMT generates higher treatment costs, which is estimated at an excess spending of $468 million within 1 year of diagnosis in the US (40). Therefore, we should adopt multidisciplinary consultation with experts and also consider the patient's treatment expectations and financial circumstances, which may make bladder cancer patients access optimal treatment.

This study has limitations due to the retrospective design of the included cases, which is easy to be affected by selection bias. Firstly, the lack of studies assessing and reporting the stage-based outcomes made it difficult to evaluate any obvious difference in survival between TMT and RC in the subgroups of patients with different stages such as T2 or >T2 tumor stage. Secondly, neoadjuvant chemotherapy may influence overall survival regardless of whether patients undergo RC or TMT. Thirdly, the papers abstracted from the database have obvious heterogeneity in their treatment methods, such as open or minimum invasive surgery for RC, and greatly differ from treatment regimen, such as radiation dose and fraction, field of irradiation, chemo agent (cisplatin use or not, combination of agent), etc. Especially, the effect of chemotherapy maybe quite different between intra-arterial infusion and systemic administration of chemo agent. So, the optimum radiation exposure and techniques in TMT need to be further investigated. Fourthly, there may be a language bias, as all included articles were published in English. Fifthly, some outcomes (any grade AEs, dose reductions) had significant heterogeneity, but they may have influenced the results. Moreover, data from the included studies were analyzed using propensity score matching to weaken the impact of treatment selection bias and potential confounding factors that are often faced in observational studies. We should note that compared to the RC group, the patients' age receiving TMT was older; therefore, the results should be interpreted carefully. Although lacking of RCTs, we believe that this meta-analysis provides valuable information for patients and clinicians.

## Conclusion

Overall, this meta-analysis demonstrates that the efficacy of TMT is non-inferior to that of RC at <10-year OS, and RC is superior to TMT at more than 10-year OS. Therefore, TMT may be a reasonable treatment option in well-selected patients who are unsuitable for surgery or are not willing to experience surgery. In the future, more high-quality, large-sample RCTs are needed to verify the results.

## Data Availability Statement

All datasets presented in this study are included in the article/ supplementary material.

## Author Contributions

HD: conceived the study, participated in its design, and coordinated and drafted the manuscript. NF and DM collected the data. HD, NF, ZN, and DM performed the statistical analysis. All authors read and approved the final manuscript.

## Conflict of Interest

The authors declare that the research was conducted in the absence of any commercial or financial relationships that could be construed as a potential conflict of interest.
